# Developing the Short‐Form of Lymphedema Needs Questionnaire for Iranian Breast Cancer Patients

**DOI:** 10.1002/cam4.70832

**Published:** 2025-03-28

**Authors:** Mozhgan Kazemzadeh, Asiie Olfatbakhsh, Sara Dorri

**Affiliations:** ^1^ Department of Statistics and Information Technology Isfahan University of Medical Sciences Isfahan Iran; ^2^ Breast Cancer Research Center Motamed Cancer Institute, ACECR Tehran Iran; ^3^ Health Information Technology Research Center Isfahan University of Medical Sciences Isfahan Iran

**Keywords:** breast cancer, breast cancer‐related lymphedema (BCRL), exploratory factor analysis (EFA), informational needs, lymphedema management, needs assessment, patient‐centered care, questionnaire development, short‐form questionnaire

## Abstract

**Purpose:**

The informational needs of patients with Breast Cancer‐Related Lymphedema (BCRL) have not been sufficiently addressed in the scientific literature. Moreover, the only existing questionnaire in this field contains many items. Therefore, the purpose of this study is to develop a short‐form version of this questionnaire.

**Methods:**

The questionnaire items were extracted from the Lymphoedema Needs Questionnaire‐Breast Cancer (LNQ‐bc). Demographic variables and clinical characteristics were considered separately. Out of 62 items, 24 were selected, and 2 additional questions were included based on feedback from 10 experts. 100 participants with BCRL completed the short‐form questionnaire. Exploratory Factor Analysis (EFA) was performed using principal components extraction and varimax rotation, and Cronbach's alpha was calculated for each dimension.

**Results:**

After evaluating the content validity, the instrument's construct validity with 26 items was conducted using EFA. The KMO (Kaiser‐Meyer‐Olkin) value was equal to 0.879 and Bartlett's sphericity test was significant (*p*‐value < 0.001), indicating the data's adequacy and appropriateness to perform EFA. Five extracted dimensions were named: “Lymphedema information needs” (5 items), “Informational support, peers” (5 items), “Access to a lymphedema care specialist” (5 items), “Physical and daily activities” (7 items) and “Financial issues and compression garments” (4 items). The level of needs in this study in all dimensions was high (more than 77%).

**Conclusions:**

The high factor loadings and the total explained variance of 78.152% support the construct validity of the short questionnaire. Although some items exhibited cross‐loadings, the majority loaded strongly on a single factor, indicating good discriminant validity. Providing services according to the needs of patients can be prioritized. Healthcare providers, insurers, and individuals should be better informed about lymphedema, its associated costs, and the importance of implementing appropriate management programs.

## 1 Introduction

Breast Cancer (bc) is the second most commonly diagnosed cancer in the world. More than 2.3 million new cases were identified in 2022. It is the leading cause of cancer‐related deaths among females worldwide. Asian populations have the highest incidence and mortality rate of this cancer [1]. Among Iranian women, BC is the most commonly diagnosed cancer. In a meta‐analysis study, the prevalence of breast cancer among Iranian women was estimated at 23.6% (95% CI: 15.3%–34.7%) [2]. In 2018, the 5‐year prevalence of breast cancer in Iran was 40,825 cases. The survival rates were 0.808, 0.695, and 0.559 in 3,5 and 10 years, respectively [3].

Various treatments, such as surgery, chemotherapy, radiotherapy, and hormone therapy, can be utilized in combination for treatment. One of the most significant complications of breast cancer treatment is lymphedema [4]. Lymphedema is a condition in which lymph fluid accumulates in tissue due to insufficient drainage [5]. Predictors of Breast Cancer Related Lymphedema (BCRL) commonly include Body Mass Index (BMI), radiotherapy, chemotherapy, and axillary lymph node dissection [6]. The level of lymph node dissection is a key determining factor, and BMI is an important patient‐related factor [7].

BCRL can lead to shoulder dysfunction, heaviness, infection, and psychological problems [8]. Evidence shows BCRL can reduce Quality of Life (QOL) [9] and impose financial burdens on patients [10]{Mobarakeh, 2019 #20}. It is estimated that in the United States, more than 40% of breast cancer survivors are affected by lymphedema [11]. Researchers estimate the prevalence of lymphedema to range from 20% to 34%, depending on the treatment modality [12]. In the Western world, the primary cause of secondary lymphedema is breast cancer treatment, occurring in approximately 20% of patients following treatment [13, 14].

In Iranian breast cancer survivors, the prevalence of lymphedema was reported to be 17.5%, with a range of 4%–21% across different study centers [15]. Early detection is essential for effective management of the condition [16]. However, many patients do not ask for help until they see noticeable edema [11]. This is often due to a lack of awareness about the possibility of developing lymphedema or the importance of preventive measures.

Various studies have acknowledged that patients do not receive adequate information about lymphedema, including how to recognize signs and symptoms, where to seek treatment and find lymphedema specialists, or how to adopt certain preventive behaviors [17, 18, 19, 20]. In Iran, 67% of patients did not receive any information about lymphedema after treatment [21]. On the other hand, scientific literature has not been sufficiently investigated regarding diagnostic methods and patient information needs [11, 18].

This lack of awareness worsens their quality of life, as lymphedema significantly impacts daily functioning and emotional well‐being [19]. These findings highlight the need for a concise tool to identify and address these gaps. By focusing on this population, our study aims to develop a tailored questionnaire that effectively captures their unique challenges. Existing tools, such as the SCNS‐SF34, often fail to address the specific needs of breast cancer survivors with lymphedema. Additionally, their length can be burdensome for patients, particularly those experiencing fatigue or cognitive difficulties. Therefore, a brief, targeted questionnaire is essential to efficiently assess and address the unmet needs of this population, ensuring more personalized care and improved outcomes.

The Lymphoedema Needs Questionnaire‐Breast Cancer (LNQ‐BC) is the proper solution with 62 questions [22]. However, considering the physical condition of these patients and their potential inability to use their hands to write, it seems logical to develop a shorter questionnaire.

Short‐form questionnaires are developed to address the drawbacks of long questionnaires, like excessive use and pressure on participants, resulting in low response rates and incomplete data [23] and are beneficial for a wider range of individuals [24]. They offer cost‐effective, easy‐to‐administer assessments [25]. The psychometric properties of short forms, such as reliability and validity, are comparable to those of long forms [26]. Short forms are particularly useful when administration time is limited, especially in research settings [27].

Considering the physical condition of these patients, their potential inability to use their hands to write, the unmet needs of this population, and the absence of a shortened questionnaire to assess their needs, we decided to develop a shortened version of the LNQ‐bc questionnaire.

## 2 Methods

### 2.1 Study Design

This was a cross‐sectional study conducted at the Motamed Cancer Institute in Tehran. This clinic is one of the dedicated breast cancer referral centers in Tehran, Iran. This study is a continuation of our previous studies on lymphedema. In the previous study, we addressed the importance of information needs, methods of information delivery, and timing of receiving necessary information about lymphedema in BCRL patients [21]. In this study, we aimed to identify the information needs of these patients.

### 2.2 Ethics Approval

The study was approved by the Executive Board of the Medical Ethics Committee of Isfahan University of Medical Sciences, with the ethical approval number IR.MUI.NUREMA.REC.1401.171. The research was conducted in accordance with the guidelines of the ethics committee, as outlined in the ethical statement and the Helsinki Declaration.

### 2.3 Data Collection

Participants were recruited from the Motamed Cancer Institute in Tehran, Iran. A total of 100 patients with confirmed Breast Cancer‐Related Lymphedema (BCRL) were included in the study. Informed consent was obtained from all participants, and they were assured that their information would remain confidential and their identities anonymous.

Several factors limited the recruitment process. Many patients experienced physical discomfort or limitations due to their lymphedema, which made it challenging for them to complete the lengthy 62‐item questionnaire. Additionally, time constraints during clinical visits further restricted the number of participants who could comfortably complete the questionnaire.

### 2.4 Inclusion/Exclusion Criteria for Participants

Participants in this study were breast cancer patients with a confirmed diagnosis of lymphedema who were referred to a specialized clinic for lymphedema treatment. For patients with limited literacy, the questionnaire was completed with the assistance of a companion, if available. Participants were permitted to withdraw from the study at any time if they experienced discomfort or chose to discontinue participation at their own discretion.

### 2.5 Questionnaire

The questionnaire items were extracted from the Lymphoedema Needs Questionnaire‐Breast Cancer (LNQ‐bc) [22]. Demographic variables and clinical characteristics were considered separately. The response of each item was considered as a 5‐point Likert scale (no need, satisfied, low need, moderate need, and high need).

### 2.6 Content Validity of the Questionnaire

The content validity of the selected items was assessed by a panel of 10 experts, chosen based on their extensive experience and expertise in BCRL. The panel included five surgeons specializing in breast cancer surgery, three lymphologists, and two physiotherapists, all with over 10 years of experience in their respective fields. The selection of experts was intentional to ensure multidisciplinary representation, covering the key areas relevant to BCRL assessment and management.

A focus group discussion was conducted to evaluate the questionnaire items. During this session, each question was thoroughly reviewed and discussed by the panel. Consensus was achieved through a structured process: initial independent scoring of each item by the experts, followed by group discussion to resolve discrepancies and refine the questions. Questions were retained, modified, or excluded based on majority agreement (defined as ≥ 80% consensus among the experts). New questions proposed during the discussion were subsequently evaluated and accepted by all experts.

### 2.7 Data Analysis

Data collected from the short‐form questionnaire were analyzed using descriptive statistics in SPSS (version 28.0; IBM). To evaluate the construct validity and extract potential dimensions of the tool, Exploratory Factor Analysis (EFA) was performed using principal component extraction and varimax rotation. The Kaiser–Meyer–Olkin (KMO) index was used to assess the adequacy of the sample. A KMO value of 0.6 or above is generally considered acceptable for factor analysis [28]. Bartlett's test of sphericity was used to examine the correlation between the questionnaire items, and its significant result confirmed the suitability of factor analysis. The variance of each dimension and the total explained variance were also calculated.

To measure the questionnaire's reliability, Cronbach's alpha was calculated for each dimension. Cronbach's alpha values > 0.9, > 0.8, > 0.7, > 0.6, > 0.5, and < 0.5 were interpreted as excellent, good, acceptable, questionable, weak, and unacceptable, respectively [29].

After conducting exploratory factor analysis and extracting and confirming dimensions, the frequency and percentage of patients' needs were calculated in two groups of patients who declared need (including low, moderate, and high needs) and patients who expressed satisfaction or lack of need.

## 3 Results

Descriptive statistics of demographic variables and clinical characteristics are presented in Table 1 and Table 2.

**TABLE 1:** Descriptive statistics of demographic variables and clinical characteristics.

Variable	Group	Frequency	Percent
Age	20–40	9	8.9
	41–60	66	61.3
	61–70	23	22.8
Education	Illiterate	7	6.9
	High school or Diploma	59	58.5
	BS	29	28.7
	MSc	3	3.0
	PhD	3	3.0
Marital status	Married	81	80.2
	Single	19	18.8
Employment status	Homemaker	68	67.3
	Working	18	17.8
	Retired	15	14.9

**TABLE 2:** Descriptive statistics of clinical characteristics.

Variable	Group	Frequency	Percent	Variable	Group	Frequency	Percent
Disease status	Undergoing treatment	14	13.9	Metastatic	Yes	10	9.9
	Recently finished treatment	28	27.7		No	47	46.5
	Recurrence diagnosis	10	9.9		Do not know	25	24.8
	Follow‐up	43	42.6	Treatment	Surgery	98	97.0
Years of treatment	< 1	8	7.9		Chemotherapy	91	90.1
	1 < < 2	8	7.9		Radiotherapy	85	84.2
	2 < < 3	11	10.9		Hormone therapy	31	30.7
	> 3	64	63.4	First‐time lymphedema diagnosis	< 3 months	14	13.9
	Undergoing treatment	9	8.9		3 < < 6 months	10	9.9
Stage of cancer	Stage I	23	31.1		6 < < 12 months	10	9.9
	Stage II	14	18.9		1 < < 2 years	15	14.9
	Stage III	6	8.1		> 2 years	48	47.5
	Stage IV	3	4				
	Do not know	28	37.8				

Most participants were middle‐aged, held diplomas, were married, and were homemakers. The majority were in follow‐up status, and more than 1 year had passed since their treatment. More than 37% of patients were unaware of their cancer stage. Additionally, for most of these patients, over 2 years had passed since their initial lymphedema diagnosis.

The content validity of the selected items was assessed by 10 experts. Twenty‐four questions were selected through the process described in the Methods section, and two new questions were added. All 26 items were approved by the experts.

After evaluating the content validity, the construct validity of the 26‐item instrument was assessed using Exploratory Factor Analysis (EFA). The Kaiser‐Meyer‐Olkin (KMO) value was 0.879, and Bartlett's test of sphericity was significant (*p* < 0.001), indicating the adequacy and suitability of the data for EFA. The initial factor analysis using Varimax rotation resulted in the extraction of three factors, which were not approved by the researchers. Subsequently, 4‐factor and 5‐factor EFA were performed, and the 5‐factor structure was ultimately approved. The five extracted dimensions were named:

1. “Lymphedema information needs” (5 items)
2. “Informational support, peers” (5 items),
3. “Access to a lymphedema care specialist” (5 items),
4. “Physical and daily activities” (7 items), and
5. “Financial issues and compression garments” (4 items).

The factor loadings of these dimensions ranged from 0.498 to 0.812, explaining 78.152% of the total variance. The Cronbach's alpha coefficient for the questionnaire was 0.969. The dimensions, along with their factor loadings, Cronbach's alpha values, and variances, are presented in Table 3.

**TABLE 3:** Dimensions, factor loadings, Cronbach's alpha, and variance of the patient needs questionnaire.

Factor	Item	Question	Factor				
			1	2	3	4	5
Lymphedema information needs	1	To be fully informed about the causes of lymphedema	0.785				
	2	To be given information about aspects of managing your lymphedema	0.812				
	3	To be adequately informed about the treatment options for lymphedema before you choose to have them	0.722				
	4	To be given information about lymphedema when first diagnosed with breast cancer	0.671				
	7	To be fully informed about techniques and activities you can do to help yourself manage lymphedema	0.554				
Informational support, peers	5	To be given access to an assessment program for early detection of lymphedema		0.598			
	6	Help with learning to feel in control of your situation		0.506			
	8	To be fully informed about lymphedema support groups in your area		0.756			
	9	To provide family members with information about lymphedema		0.615			
	10	To be informed of the availability of lymphedema treatment centers		0.545			
Access to lymphedema care specialist	11	To receive adequate information about lymphedema from your doctor specifically			0.599		
	12	To be referred to a specifically trained lymphedema physiotherapist			0.692		
	13	Having doctor(s) who are fully informed about lymphedema and its associated problems			0.804		
	14	Having doctor(s)/ health care professionals willing to follow up with your lymphedema treatment			0.747		
	15	Having health care professionals (e.g., nurses) fully informed about lymphedema			0.509		
Physical and daily activities	16	Coping with work around the home				0.675	
	17	Dealing with reduced physical mobility because of lymphedema				0.685	
	18	Help with overcoming difficulties with daily activities because of the lymphedema				0.72	
	19	Availability of clothes to hide the arm				0.554	
	20	To be given information about problems with sexual relationships that may arise because of the lymphedema				0.658	
	24	concern about doing suitable exercises to lose weight				0.731	
	25	concern about doing suitable exercises to improve lymphedema				0.77	
Financial issues and Garments	21	Availability and variety of garments required for the treatment of lymphedema					0.756
	22	Coping with problems with garments required for the treatment of lymphedema					0.737
	23	Wanting more information about finding garments and other treatment needs for lymphedema					0.73
	26	Dealing with concerns about your financial situation because of the costs involved with lymphedema					0.498
		Variance	57.61	7.76	5.39	3.83	3.56
		Total variance explained	78.15				
		Cronbach's alpha	0.899	0.866	0.945	0.915	0.900
		Cronbach's alpha of questionnaire	0.969				

The high factor loadings (ranging from 0.498 to 0.812) and the total explained variance of 78.152% support the construct validity of the questionnaire. Although some items exhibited cross‐loadings, the majority loaded strongly on a single factor, indicating good discriminant validity.

**FIGURE 1:**
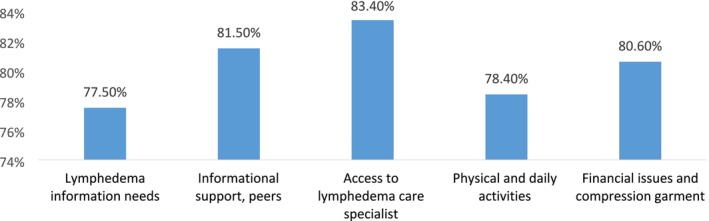
Percent of declaring need in 5 extracted factors.

For each of the factors, the frequency and percentage of patients' needs were calculated for two groups: (1) patients who reported a need (including low, moderate, and high need) and (2) patients who expressed satisfaction or no need. The rate of reported needs across the five extracted dimensions ranged from 77.5% to 83.4%, indicating a high level of unmet needs among patients in all areas of the study. The highest support needs of patients, in descending order, were: “Access to a lymphedema care specialist” (83.4%), “Informational support, peers” (81.5%), “Financial issues and compression garments” (80.6%), “Physical and daily activities” (78.4%), and “Lymphedema information needs” (77.5%) (Table 4 and Figure 1).

**TABLE 4:** Frequency and percent of total responses in 5 extracted factors.

Factor	Name	No of items	Satisfaction or lack of need satisfaction		Low, moderate or high need		Total
			Frequency	Percent	Frequency	Percent	
1	Lymphedema information needs	5	103	22.5%	355	77.5%	458
2	Informational support, peers	5	84	18.5%	371	81.5%	455
3	Access to lymphedema care specialist	5	76	16.6%	383	83.4%	459
4	Physical and daily activities	7	139	21.6%	504	78.4%	643
5	Financial issues and compression garments	4	73	19.4%	303	80.6%	376
Total		26	475	19.9%	1916	80.1%	2391

Then, in each factor, the percentage of declaration of needs (low, moderate, and high needs) was calculated in response to each question.

The “Lymphedema information needs” factor highlights patients' need for comprehensive information about the causes of lymphedema, treatment options, and self‐management techniques. Patients expressed a strong desire to be informed about lymphedema at the time of their initial breast cancer diagnosis. This underscores the importance of early patient education to prevent lymphedema or manage it effectively if it occurs. The percentage of reported needs varied from 70% to 89.5%. The items “*To be fully informed about techniques and activities you can do to help yourself manage lymphedema*” and “*To be fully informed about the causes of lymphedema*” had the highest (89.4%) and lowest (69.9%) percentages, respectively (Table 5).

**TABLE 5:** Frequency and percent of the responses in factor 1.

Item	Question	Satisfaction or lack of need satisfaction		Low, moderate or high need		Total
		Frequency	Percent	Frequency	Percent	
1	To be fully informed about the causes of lymphedema	28	30.1%	65	69.9%	93
2	To be given information about aspects of managing your lymphedema	23	25.3%	68	74.7%	91
3	To be adequately informed about the treatment options for lymphedema before you choose to have them	20	22.7%	68	77.3%	88
4	To be given information about lymphedema when first diagnosed with breast cancer	22	23.9%	70	76.1%	92
7	To be fully informed about techniques and activities you can do to help yourself manage lymphedema	10	10.6%	84	89.4%	94
	Total	103	22.5%	355	77.5%	458

The “Informational support, peers” factor emphasizes the need for access to early detection programs, support groups, and information about treatment centers. Patients also expressed a desire for their family members to be informed about lymphedema. This highlights the critical role of social and emotional support in managing lymphedema. In this factor, the percentage of reported needs ranged from 71.4% to 86%. The item “Help with learning to feel in control of your situation” had the highest percentage (86%), while “To be fully informed about lymphedema support groups in your area” had the lowest percentage (71.4%) of reported needs (Table 6).

**TABLE 6:** Frequency and percent of responses in factor 2.

Item	Question	Satisfaction or lack of need satisfaction		Low, moderate or high need		Total
		Frequency	Percent	Frequency	Percent	
5	To be given access to an assessment program for early detection of lymphedema	13	14.4%	77	85.6%	90
6	Help with learning to feel in control of your situation	11	12.0%	81	88.0%	92
8	To be fully informed about lymphedema support groups in your area	26	28.6%	65	71.4%	91
9	To provide family members with information about lymphedema	21	23.3%	69	76.7%	90
10	To be informed of the availability of lymphedema treatment centers	13	14.1%	79	85.9%	92
	Total	84	18.5%	371	81.5%	455

In the “Access to a lymphedema care specialist” factor, patients expressed a need for access to healthcare professionals who are knowledgeable about lymphedema and its associated complications. They preferred to be referred to specifically trained lymphedema physiotherapists and to receive follow‐up care from them. This highlights the importance of specialized care in the treatment journey. In this factor, the percentage of reported needs in all questions was approximately the same (varied between 81.7% and 84.8%). Two items “*To be referred to a specifically trained lymphedema physiotherapist*” and “*Having doctor(s)/ healthcare professionals willing to follow up with your lymphedema treatment*” had the highest percentage (84.8%) and the item “*Having health care professionals (e.g. nurses) fully informed about lymphedema*” had the lowest (81.7%) percentage reported needs in this area (Table 7).

**TABLE 7:** Frequency and percent of responses in factor 3.

Item	Question	Satisfaction or lack of need satisfaction		Low, moderate or high need		Total
		Frequency	Percent	Frequency	Percent	
11	To receive adequate information about lymphedema from your doctor specifically	16	17.80%	74	82.20%	90
12	To be referred to a specifically trained lymphedema physiotherapist	14	15.20%	78	84.80%	92
13	Having doctor(s) who are fully informed about lymphedema and its associated problems	15	16.30%	77	83.70%	91
14	Having doctor(s)/ health care professionals willing to follow up with your lymphedema treatment	14	15.20%	78	84.80%	90
15	Having health care professionals (e.g. nurses) fully informed about lymphedema	17	18.30%	76	81.70%	92
	Total	76	16.56%	383	83.44%	459

“Physical and daily activities” factor focuses on the challenges patients face in performing daily activities due to lymphedema. Patients also expressed a need for information about suitable exercises to improve their condition and lose weight. This highlights the necessity for tailored physical activity programs to enhance patients' quality of life. The percentage of reported needs varied between 64.5% and 84.8%. Two items “concern about doing suitable exercises to lose weight” and “concern about doing suitable exercises to improve lymphedema” respectively had the highest percentage, and two items “*To be given information about problems with sexual relationships that may arise because of the lymphedema*” and “*Availability of clothes to hide the arm*” respectively had the lowest percentage of declaration of need in this area (Table 8).

**TABLE 8:** Frequency and percent of responses in factor 4.

Item	Question	Satisfaction or lack of need satisfaction		Low, moderate or high need		Total
		Frequency	Percent	Frequency	Percent	
16	Coping with work around the home	18	19.6%	74	80.4%	92
17	Dealing with reduced physical mobility because of lymphedema	19	20.9%	72	79.1%	91
18	Help with overcoming difficulties with daily activities because of the lymphedema	17	18.7%	74	81.3%	91
19	Availability of clothes to hide arm	22	23.9%	70	76.1%	92
20	To be given information about problems with sexual relationships that may arise because of the lymphedema	33	35.5%	60	64.5%	93
24	Concern about doing suitable exercises to lose weight	16	17.4%	76	82.6%	92
25	Concern about doing suitable exercises to improve lymphedema	14	15.2%	78	84.8%	92
	Total	139	21.6%	504	78.4%	643

In the “Financial issues and compression garments” factor, patients reported concerns about the availability and cost of compression garments, as well as other treatment‐related expenses. This factor highlights the financial burden associated with lymphedema treatment and underscores the need for better insurance coverage and financial support. The percentage of reported needs ranged from “77.7% to 83.3%”. The item “*Dealing with concerns about your financial situation because of the costs involved with lymphedema*” had the highest percentage (83.3%), while the item “*Availability and variety of garments required for the treatment of lymphedema*” had the lowest percentage (77.7%) in this area (Table 9).

**TABLE 9:** Frequency and percent of responses in factor 5.

Item	Question	Satisfaction or lack of need satisfaction		Low, moderate or high need		Total
		Frequency	Percent	Frequency	Percent	
21	Availability and variety of garments required for the treatment of lymphedema	21	22.3%	73	77.7%	94
22	Coping with problems with garments required for the treatment of lymphedema	18	19.4%	75	80.6%	93
23	Wanting more information about finding garments and other treatment needs for lymphedema	18	19.4%	75	80.6%	93
26	Dealing with concerns about your financial situation because of the costs involved with lymphedema	16	16.7%	80	83.3%	96
	Total	73	19.4%	303	80.6%	376

## 4 Discussion

In this study, factor analysis was conducted, and five factors were identified as the best fit. Internal reliability was assessed as excellent, and the high factor loadings, along with the total explained variance of 78.15%, support the construct validity of the questionnaire. In this study, 5 dimensions of the questionnaire were explored. “Lymphedema information needs” (5 items), “Informational support, peers” (5 items), “Access to a lymphedema care specialist” (5 items), “Physical and daily activities” (7 items), and “Financial issues and compression garments” (4 items).

In Taleghani's study, the first essential aspect of empowering bc patients was timely and comprehensive information [30]. In the current study, the first factor, patients expressed a desire to be fully informed about the causes of lymphedema, treatment options, and self‐management strategies. Patient education focusing on prevention and early symptom recognition is essential for effectively managing lymphedema [31]. Education about lymphedema before breast cancer treatment is crucial to prevent its development [32]. Improved patient education has been shown to reduce lymphedema symptoms after surgery [33].

Our findings on patient education align with established research. Fu and White emphasize the role of education in improving lymphedema outcomes, which is consistent with our focus on providing comprehensive information about the causes, treatment, and self‐management of breast cancer‐related lymphedema [18, 19].

The second factor of the questionnaire highlighted the importance of supporting groups. This includes assessment programs for the early detection of lymphedema, access to treatment centers and peer groups, strategies for managing the condition, and providing information to family members.

Support programs for bc survivors should be integrated into survivorship care programs, especially for those who struggle to express their Breast Cancer‐Related Lymphedema (BCRL) symptoms to others [34]. Support for lymphedema patients with breast cancer is crucial for their self‐management and overall well‐being. Studies have highlighted the significance of factors such as self‐efficacy, social support, knowledge, and self‐regulation in enhancing lymphedema self‐management behaviors among breast cancer survivors [35, 36].

Furthermore, interventions focusing on strengthening self‐efficacy and social support have been identified as key components in enhancing self‐care abilities and reducing the burden of lymphedema treatment costs for patients [37].

Access to lymphedema care specialists is another critical factor. Patients expressed a desire to receive information and care from doctors who are knowledgeable about Breast Cancer‐Related Lymphedema (BCRL) and its associated complications. They also preferred to be referred to specifically trained lymphedema physiotherapists and to receive follow‐up care from them. Access to healthcare specialists plays a vital role in the treatment journey.

Zhao stated that self‐management plays a vital role in BCRL therapy, with patients and healthcare professionals emphasizing the need for knowledge, emotional support, and healthcare provider training [38]. Dedicated treatment centers for lymphedema in Iran are still limited. Many patients do not refer to specialized centers for treatment and are missing the opportunity to receive timely care. Many patients are unaware of where to seek help when they develop lymphedema [39].

Ensuring access to a multidisciplinary team of specialists is vital for addressing the complex needs of BCRL patients and improving outcomes. Professionals involved in lymphedema care play a crucial role in supporting patients with BCRL [38]. Furthermore, the provision of supportive care, both from healthcare professionals and family members, can significantly improve the physical and psychosocial well‐being of women with breast cancer‐related lymphedema [40]. Today, technology can be utilized to screen for lymphedema in a reliable, valid, and cost‐effective manner [41].

The “Physical and daily activities” factor focused on work, physical activity, mobility, and sexuality. A key concern for patients was identifying suitable exercises to lose weight or improve their health.

Breast cancer diagnosis and treatment can lead to physical, social, and psychological challenges, significantly impacting post‐treatment recovery [42]. Studies have shown that physical activity can help reduce lymphedema volume, decrease pain intensity, and improve emotional/mental well‐being and body image [43, 44]. Supervised aerobic and resistance exercises, when tailored to individual needs, positively impact physical and mental symptoms of BCRL without long‐term adverse effects [45].

The last factor was financial issues and garments. Availability, finding garments, and management of its problems are the important questions of patients. Patients expressed concerns about the availability and selection of compression garments, as well as managing related challenges.

BCRL imposes a significant financial burden on patients and society. Studies show that BCRL treatment costs are substantial, and patients often have to cover the expenses out of pocket [46]. In the United States, breast cancer survivors with lymphedema face 122% higher mean overall monthly direct costs compared to those without lymphedema, with elevated costs persisting over time due to inadequate insurance coverage and socioeconomic disparities [47]. Unfortunately, post‐treatment services are neglected due to the high cost of treatment in many countries. Therefore, policymakers and healthcare providers must take effective actions to reduce the financial burden of the costs.

Ridner's study showed that bc survivors with lymphedema face challenges in finding adequate garments due to a lack of resources and support [48]. Prioritizing patients' needs can help researchers focus on the specific information and solutions required to address these challenges effectively.

The highest support needs of patients in our study were, in order “Access to a lymphedema care specialist”, “informational support, peers”, “Financial issues and compression garments”, “physical and daily activities” and “Lymphedema information needs”.

Unmet needs can vary from person to person, and demographic factors may influence these needs. The COVID‐19 pandemic has significantly impacted breast cancer‐related lymphedema (BCRL). It may reduce the effectiveness of BCRL treatment [49], as delays in cancer treatment can pose risks for patients [50].

In Iran, lymphedema and its associated challenges remain largely unrecognized. There is a need for greater insurance coverage and increased awareness, particularly among healthcare professionals and breast cancer patients before surgery.

## 5 Conclusion

The short‐form questionnaire, with its strong construct validity, can be used to identify and prioritize the needs of patients, enabling the provision of tailored services. This study highlights the importance of addressing informational, physical, and financial needs in the management of Breast Cancer‐Related Lymphedema (BCRL). While the findings align with existing literature, their generalizability may be limited to the Iranian population. Future studies should validate the tool across diverse cultural contexts. Additionally, barriers such as lack of awareness, resource constraints, and low patient engagement must be addressed. Healthcare providers, insurers, and individuals should be better informed about lymphedema and its associated costs, and appropriate prevention programs should be implemented.

### 5.1 Healthcare Policy and Practice Implications

1. Integrate lymphedema education into breast cancer care.
2. Develop support programs and train healthcare professionals.
3. Expand insurance coverage for lymphedema treatment.

### 5.2 Limitations

This study has several limitations. First, the absence of a direct comparison between the short‐ and long‐form questionnaires hinders a comprehensive evaluation of their trade‐offs. Second, single‐center recruitment from a specialized Tehran‐based institute may limit generalizability, particularly for rural or low‐resource populations. Multi‐center studies with diverse cohorts are needed to validate the tool. Third, the small sample size (*n* = 100) may reduce the robustness of exploratory factor analysis (EFA); a larger sample would enhance reliability and allow subgroup analyses. Finally, the lack of qualitative feedback on the questionnaire's usability and relevance represents a missed opportunity to refine the tool based on patient perspectives.

### 5.3 Future Study and Next Steps

Future studies should include direct comparisons between the short‐ and long‐form questionnaires to assess whether the short form captures the same depth of information as the original version. Incorporating patient perspectives is essential to ensure the questionnaire's relevance and usability. Additionally, refining items with significant cross‐loadings will enhance the tool's clarity and discriminant validity. Validation in diverse clinical settings, including rural and low‐resource areas, is crucial to ensure broader applicability. Longitudinal, intervention, and comparative studies with larger sample sizes are needed to further validate the tool and improve patient outcomes. Combining quantitative findings with qualitative interviews could provide deeper insights into the consistency and comprehensiveness of identified needs. Finally, examining the underlying factors influencing these needs may offer valuable directions for future research.

## Author Contributions

M.K., A.O., and S.D. contributed to the study concept and design. Data acquisition was done by S.D. and A.O. Statistical analysis was done by M.K. The first draft of the manuscript was written by S.D., and all authors commented on previous versions of the manuscript. All authors read and approved the final manuscript.

## Ethics Statement

We received the ethical number IR.MUI.NUREMA.REC.1401.171 from the Executive Board of the Medical Ethics Committee of the Isfahan University of Medical Sciences.

## Consent

Informed consent was obtained from all participants, and they were assured that their information would remain confidential and their identities anonymous.

## Conflicts of Interest

The authors declare no conflicts of interest.

## Data Availability

Data are available upon reasonable request.
